# Changes in the microbial consortium during dark hydrogen fermentation in a bioelectrochemical system increases methane production during a two-stage process

**DOI:** 10.1186/s13068-018-1175-z

**Published:** 2018-06-22

**Authors:** Kengo Sasaki, Daisuke Sasaki, Yota Tsuge, Masahiko Morita, Akihiko Kondo

**Affiliations:** 1 Graduate School of Science, Technology and Innovation, Kobe University, 1-1 Rokkodaicho, Nada-ku, Kobe, Hyogo 657-8501 Japan; 2 Institute for Frontier Science Initiative, Kanazawa University, Kakuma-machi, Kanazawa, Ishikawa 920-1192 Japan; 3 Environmental Chemistry Sector, Environmental Science Research Laboratory, Central Research Institute of Electric Power Industry, 1646 Abiko, Abiko-shi, Chiba-ken 270-1194 Japan; 4 RIKEN Center for Sustainable Resource Science, 1-7-22 Suehiro-cho, Tsurumi-ku, Yokohama, Kanagawa 230-0045 Japan

**Keywords:** Bioelectrochemical system, Dark fermentation, Hydrogen, Methane, Microbial consortium, Two-stage process

## Abstract

**Background:**

Bioelectrochemical systems (BESs) are an innovative technology developed to influence conventional anaerobic digestion. We examined the feasibility of applying a BES to dark hydrogen fermentation and its effects on a two-stage fermentation process comprising hydrogen and methane production. The BES used low-cost, low-reactivity carbon sheets as the cathode and anode, and the cathodic potential was controlled at − 1.0 V (vs. Ag/AgCl) with a potentiostat. The operation used 10 g/L glucose as the major carbon source.

**Results:**

The electric current density was low throughout (0.30–0.88 A/m^2^ per electrode corresponding to 0.5–1.5 mM/day of hydrogen production) and water electrolysis was prevented. At a hydraulic retention time of 2 days with a substrate pH of 6.5, the BES decreased gas production (hydrogen and carbon dioxide contents: 52.1 and 47.1%, respectively), compared to the non-bioelectrochemical system (NBES), although they had similar gas compositions. In addition, a methane fermenter (MF) was applied after the BES, which increased gas production (methane and carbon dioxide contents: 85.1 and 14.9%, respectively) compared to the case when the MF was applied after the NBES. Meta 16S rRNA sequencing revealed that the BES accelerated the growth of *Ruminococcus* sp. and Veillonellaceae sp. and decreased *Clostridium* sp. and *Thermoanaerobacterium* sp., resulting in increased propionate and ethanol generation and decreased butyrate generation; however, unknowingly, acetate generation was increased in the BES.

**Conclusions:**

The altered redox potential in the BES likely transformed the structure of the microbial consortium and metabolic pattern to increase methane production and decrease carbon dioxide production in the two-stage process. This study showed the utility of the BES to act on the microbial consortium, resulting in improved gas production from carbohydrate compounds.

**Electronic supplementary material:**

The online version of this article (10.1186/s13068-018-1175-z) contains supplementary material, which is available to authorized users.

## Background

Bioelectrochemical systems (BESs) are a novel technology based on both biological and electrochemical processes for the production of valuable products from waste and wastewater [1–3]. BESs consist of an anode and cathode, and can change microbial fermentation by overcoming the thermodynamic limits of metabolic routes [4]. Anaerobic digestion is a classic method of degrading complex molecules such as waste materials into methane and carbon dioxide via a mixed microbial consortium [5, 6], which offers benefits such as the lack of need for sterilisation for microbial processing of waste streams and resilience to adverse conditions [5]. BESs have been used in anaerobic digestion of organic matter to influence the structure and/or metabolism of the microbial consortium and boost hydrogen production [7], methane production [8–10], and biohythane production [11].

Gaseous fuels such as hydrogen and methane, which can be used in heat and/or electricity production and for transport, are often obtained via two-stage fermentation [12, 13]. In the first stage, organic matter in the substrate is degraded to release hydrogen during dark fermentation. In the second stage, residual carbohydrates such as volatile fatty acids and alcohols are further converted into methane. Compared with single-stage methane fermentation, the two-stage fermentation process has advantages of high energy production, a high organic loading rate (OLR), and a stable process for optimising process parameters and breaking down inhibitors in the first stage [12]. We previously used a BES to apply a potential in hydrogen fermentation [7]. The configuration of this system was similar to that of a single-chamber microbial electrolysis cell (MEC) without a membrane [14]. However, it differed in the following ways. Our BES used low-cost carbon sheets (graphite blocks) as the working and counter electrodes and a high anode potential (e.g., 1.61 V vs. Ag/AgCl) was produced using a potentiostat to inhibit methanogenic activity and prevent hydrogen consumption [15]. Moreover, our BES enabled hydrogen fermentation from artificial garbage slurry at relatively high pH conditions of 5.5–7.2 [7]. In general, the pH during dark hydrogen fermentation is 5.0–7.0; however, relatively high pH conditions are favoured to prevent accidental decreases in hydrogen production due to the accumulation of by-products (i.e., acids) and a decreased pH [16]. Regardless, the detailed effects of a BES on polarising electrodes in the microbial consortium in hydrogen fermentation are unclear. However, the effects of a BES on the microbial consortium in other environments (e.g., methane fermentation) have been investigated, and it has been shown to activate methanogenic archaea and/or enhance direct interspecies electron transfer between microorganisms, increasing methane generation [10, 17]. In addition, research on the two-stage approach involving bioelectrochemical hydrogen fermentation and methane fermentation is limited [7].

The aim of this study is to investigate the effects of a BES for polarising electrodes on the microbial consortium in the first stage of fermentation (i.e., hydrogen fermentation) at a pH greater than 6.0 using glucose as the model organic substrate. Next-generation sequencing was employed to characterise the change in the composition of the microbial consortium after the use of the BES. In the second stage, a methane fermenter (MF) was applied and its effects were evaluated using a packed-bed reactor containing carbon fibre textiles (CFTs) as supporting material for retaining microorganisms, because packed-bed systems are capable of increasing the OLR and methanogenesis [18].

## Methods

### Configuration and operation of the BES for hydrogen fermentation

**Fig. 1:**
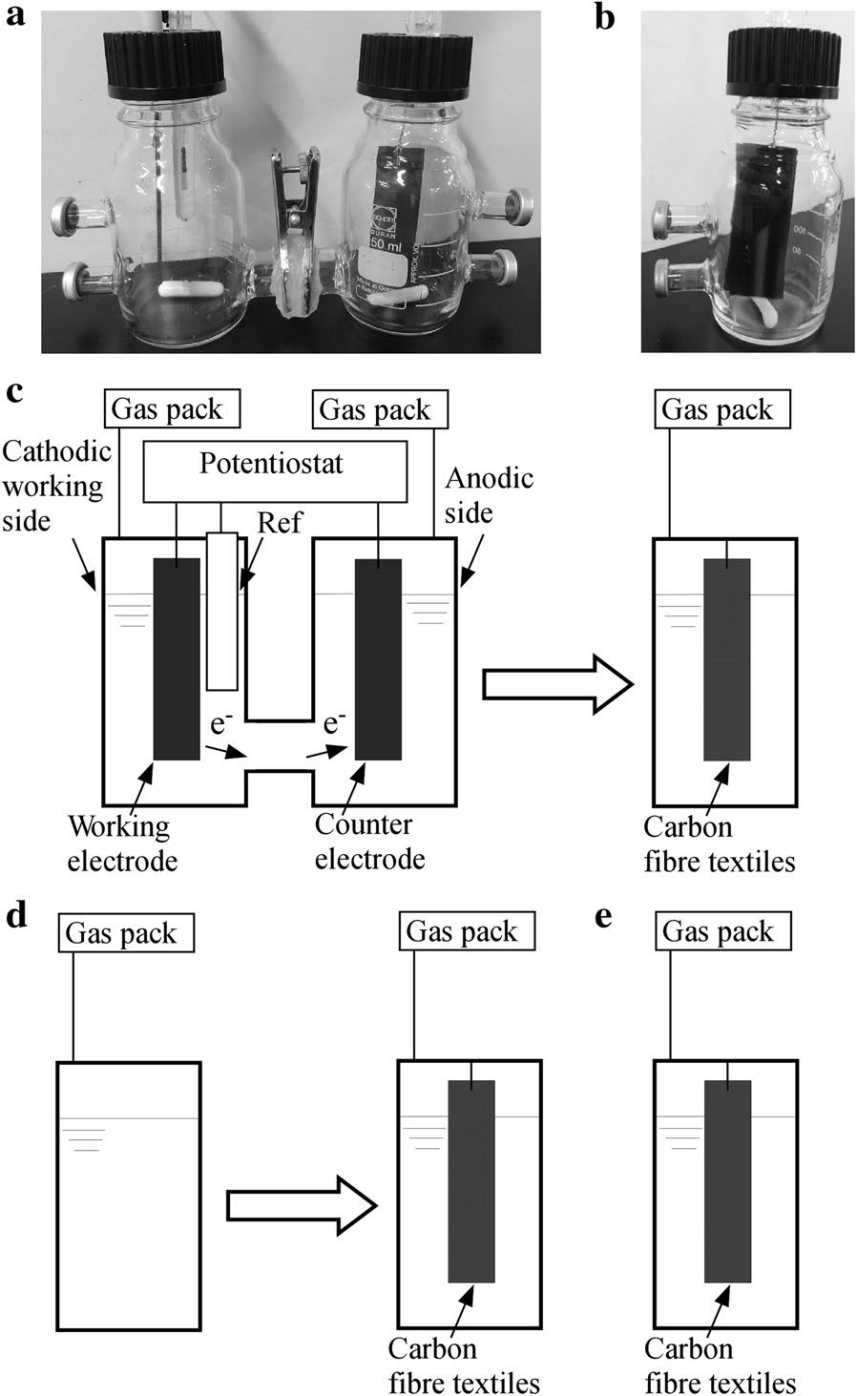
Photograph of the **a** bioelectrochemical system (BES) and **b** packed-bed system for the methane fermenter (MF). Schematic of the **c** two-stage process including BES and MF, **d** two-stage process including the non-bioelectrochemical system (NBES) without an electrode and MF, and **e** single-stage MF

For the first stage of hydrogen fermentation, an H-type two glass reactor (working volume of each reactor: 250 mL; total working volume: 500 mL) for the BES was constructed, as described previously [7]. Both the working electrode (cathode) and counter electrode (anode) were composed of carbon sheets (graphite blocks with dimensions of 25 × 75 × 2 mm) (Fig. 1a). An Ag/AgCl reference electrode (saturated KCl) was inserted in the cathodic working side. All potentials reported here are with respect to that of the Ag/AgCl reference electrode (199 mV vs. that of a standard hydrogen electrode). The potential of the working electrode was electrochemically regulated to − 1.0 V (vs. Ag/AgCl) using a potentiostat (PS-08; Tohogiken, Japan). Then, 500 mL of sludge from the methane fermenter (55 °C) to degrade 1% glucose was used to inoculate the BES. Each glass reactor had two medium/sludge sampling ports and one gas outlet port connected to a gas sampling collection bag. One glass reactor without an electrode (250 mL) was used as the control [described as a non-bioelectrochemical system (NBES)]. As described above, 250 mL of sludge was used to inoculate the NBES. The contents of both the cathodic working side and anodic counter side in the BES and NBES were mixed thoroughly using a magnetic stirrer. The temperature of the culture was maintained at 55 °C. The operation was performed in duplicate.

The BES and NBES were fed with an artificial medium. The medium (per liter) was composed of glucose: 10 g, KH_2_PO_4_: 0.1 g, K_2_HPO_4_: 0.2 g, yeast extract: 1 g, NaHCO_3_: 2 g, NH_4_Cl: 1 g, MgCl_2_·6H_2_O: 0.1 g, CaCl_2_·2H_2_O: 0.1 g, NaCl: 0.6 g, trace element solution (Deutsche Sammlung von Mikroorganismen und Zellkulturen [DSMZ] 141 medium): 10 mL, and vitamin solution (DSMZ 141 medium): 1 mL. Once per day, a predetermined volume of the fermentation liquid on both the cathodic and anodic sides of the BES and control reactor was discharged and the same volume of fresh artificial medium was added. The hydraulic retention time (HRT) was 2 or 4 days, meaning that half or one-fourth of the fermentation liquid was exchanged daily.

### Configuration and operation of the pecked-bed reactor for methane fermentation

One glass reactor (250 mL) was used for the second stage of methane fermentation (Fig. 1b). Each glass reactor was packed with two sheets of CFT (type: pitch; porosity: ~ 98%; diameter: 25.0 mm; height: 70.0 mm; thickness: 2.4 mm) as support material. The inoculum and operational temperature were the same as those in the first stage. Once per day, a predetermined amount of fermentation liquid was discharged and the same volume of the effluent from the BES or NBES was added. The effluents of both the cathodic and anodic sides of the BES (total working volume of 500 mL) were mixed and half of this mixture was added to the second stage (working volume of 250 mL) (Fig. 1c). The effluent of the NBES was added directly to the second stage (Fig. 1d). In addition, the CFT-packed reactor was operated in a single stage of the MF by adding the above-mentioned artificial medium (Fig. 1e).

### Analysis of reactor performance

The volume of gas produced was measured with a water displacement method using a graduated cylinder. The methane, carbon dioxide, and hydrogen contents of the gas were measured using a gas chromatograph equipped with a thermal conductivity detector (GC390B; GL Sciences, Tokyo, Japan) and stainless-steel column packed with active carbon (30/60 mesh; GL Sciences). The soluble total organic carbon (S-TOC) in the culture was analyzed using the HACH method (HACH Co., Loveland, Co., USA). The concentrations of lactate, acetate, propionate, butyrate, and ethanol were measured using high-pressure liquid chromatography (Shimadzu, Kyoto, Japan) with an Aminex HPX-87H column (Bio-Rad Laboratories, Hercules, CA, USA) and RID-10A refractive index detector (Shimadzu). The operation was performed at 65 °C using 5 mM H_2_SO_4_ as the mobile phase at a flow rate of 0.6 mL/min. The suspension was filtered through a glass fibre filter (0.45 μm) to determine the cell mass in the culture, the residue on the fibre was dried at 105 °C for 120 min, and the dry weight was measured. The carbon content in the cell mass was calculated using an empirical formula (C_5_H_7_NO_2_) for a microbial cell [19].

### Isolation of bacterial and archaeal DNA

Whole genomic DNA from the culture was prepared as follows. A 5000-μL aliquot of each culture was centrifuged at 5000×*g* and pelleted material was suspended in 200 μL of Tris-EDTA buffer (10 mM Tris-HCl, 1 mM EDTA, pH 8.0). This suspended material was transferred to a sterilised and DNA-free bead-beating tube containing 300 mg of glass beads (diameter: 0.1 mm). Approximately 500 μL of Tris-EDTA-saturated phenol, 250 μL of lysis buffer, and 50 μL of 10% (w/v) sodium dodecyl sulphate were added to each tube. Then, the mixture was shaken vigorously for 30 s at 5.0 m/s using a FastPrep-24 instrument (MP Biomedicals, USA). Next, the mixture was centrifuged at 22,000×*g* for 5 min. The upper aqueous layer was transferred to a fresh tube containing 275 μL of isopropyl alcohol and a 1/10 volume of 3 M sodium acetate, and chilled at − 20 °C for 10–15 min. The extracted DNA precipitate was pelleted by centrifugation at 22,000×*g* for 5 min, washed with 70% ethanol, and then dried under a vacuum. The DNA subsequently was dissolved in Tris-EDTA buffer.

### Illumina library generation

The V3–V4 region of the prokaryotic 16S rRNA gene was amplified using Pro341F (5′-CCTACGGGNBGCWSCAG-3′) and Pro805R (5′-GACTACNVGGGTATCTAATCC-3′) [20], where N, B, W, and V correspond to degenerate nucleotides A/C/G/T, G/T/C, A/T, and A/C/G, respectively. Illumina adapter overhang nucleotide sequences were added to the gene-specific sequences. The PCR reaction and amplicon pool preparation were performed according to the manufacturer’s instructions (Illumina, San Diego, CA, USA). Each PCR reaction used 12.5 ng of template DNA, along with 200 nM of each primer, and 12.5 μL of KAPA HiFi HotStart ReadyMix (KAPA Biosystems, Massachusetts, USA). The PCR reaction conditions were as follows: initial denaturation at 95 °C for 3 min; 25 cycles at 95 °C for 30 s, 55 °C for 30 s, and 72 °C for 30 s; and a final extension at 72 °C for 5 min. The amplicons were purified using the Agencourt AMPure XP beads (Beckman Coulter, Inc., California, USA). The pooled 16S rRNA gene products (5 nM) [along with an internal control (PhiX control V3; Illumina)] were subjected to paired-end sequencing using a MiSeq sequencer (Illumina) with a 600-cycle MiSeq reagent kit (Illumina). The PhiX sequences were removed and paired-end reads with *Q* scores of ≥ 20 were joined using the QIIME version 1.9.1 software package [21]. The UCLUST algorithm [22] was used to cluster filtered sequences into operational taxonomic units (OTUs) based on a 97% similarity threshold. Chimeric sequences were detected and excluded from the library using USEARCH [22]. Representative sequences from each OTU were taxonomically classified via the GreenGenes taxonomic database using the Ribosomal Database Project (RDP) classifier [23]. The OTUs were used for alpha-diversity estimation of the Shannon–Wiener diversity. Principal coordinate analysis (PCoA) was conducted using OTU information from each sample and calculated based on unweighted UniFrac distances using QIIME. All raw sequence data generated in this study are stored in MG-RAST as “Two-stage hydrogen/methane fermentation process with bioelectrochemical system” under the Accession Numbers mgm4779451.3–mgm4779466.3.

## Results

### Inhibitory effect of electrochemical regulation on methanogenesis at a relatively low organic load

The effect of electrode polarisation on reactor performance was investigated at a relatively low OLR, 1103.8 mg-C/L/day, corresponding to 13.9 mM_glucose_/L/day, and an HRT of 4 days (Table 1). Three types of reactors were operated, those with two-stage processes of BES → MF containing CFT and NBES → MF containing CFT, and a single-stage MF containing CFT (Fig. 1). Methanogenesis was inhibited in the BES and NBES by loading the artificial medium at a pH of 6.5, which contained glucose as the major carbon source, and the pH was maintained at ≥ 6.2 (Table 1). The potential of the working electrode in the BES was regulated to − 1.0 V (vs. Ag/AgCl). Under standard conditions (25 °C, pH = 7), hydrogen is formed via electrolysis of water under − 414 mV (vs. the standard hydrogen electrode). However, no abiotic hydrogen production occurred when the less-reactive carbon sheet (i.e., graphite block) was used as the electrode.

**Table 1:** Operation conditions of the two-stage and single-stage fermentation process

Reactor type	OLR	HRT	pH_in_, pH_out_
	mg-C/L/day	day	
Two-stage			
BES → MF	1103.8 → 793.3	4 → 4	6.5, 6.2 → 6.2, 7.9
BES → MF	2207.6 → 1656.0	2 → 4	6.5, 6.2 → 6.2, 7.7
BES → MF	2207.6 → 1616.3	2 → 4	7.3, 6.1 → 6.1, 7.7
NBES → MF	1103.8 → 707.7	4 → 4	6.5, 6.5 → 6.5, 7.8
NBES → MF	2207.6 → 1507.7	2 → 4	6.5, 5.9 → 5.9, 7.8
NBES → MF	2207.6 → 1533.5	2 → 4	7.3, 5.7 → 5.7, 7.7
Single-stage			
MF	1103.8	4	7.7, 7.2

Two-stage process: (bioelectrochemical system (BES) → methane fermenter (MF) containing carbon fibre textiles (CFTs) or non-bioelectrochemical system (NBES) → MF containing CFTs)

Single-stage fermentation process: (MF containing CFTs)

**Fig. 2:**
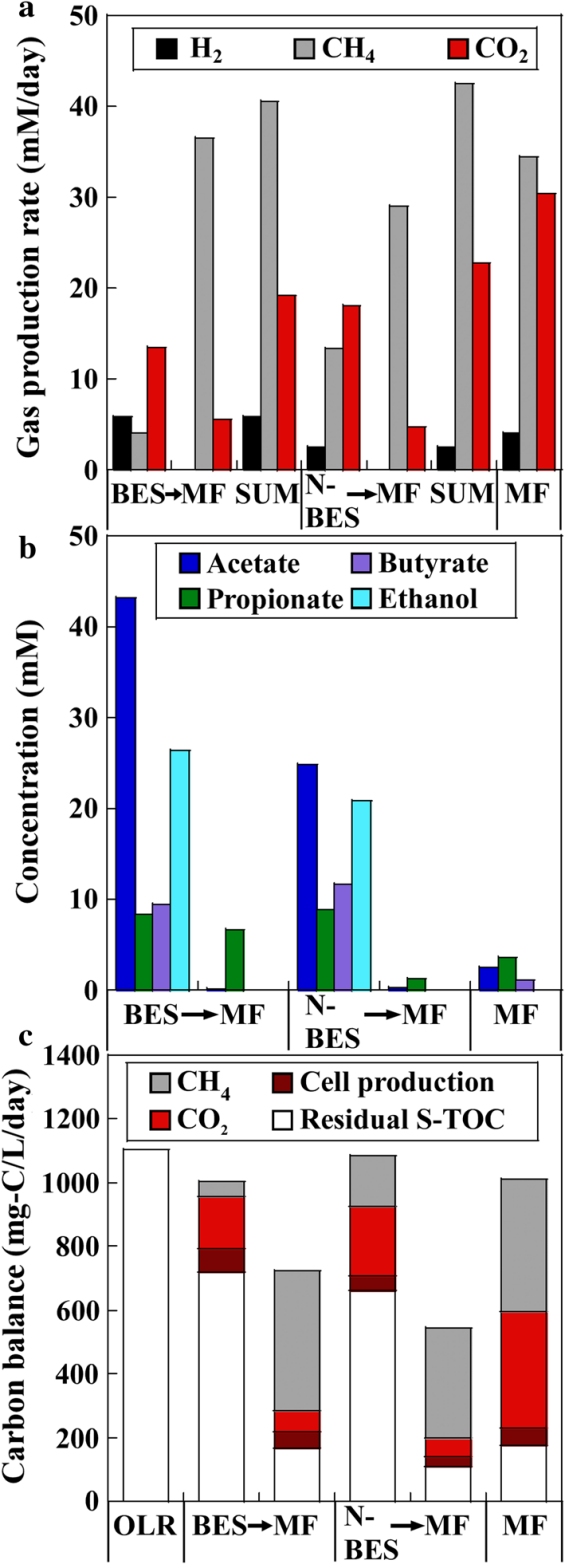
Reactor performances of the two-stage processes of bioelectrochemical system (BES) → methane fermenter (MF) and non-bioelectrochemical system (NBES) → methane fermenter (MF), and single-stage process of methane fermenter (MF). **a** Rate of gas production of hydrogen, methane, and carbon dioxide, **b** productions of soluble metabolic products. **c** Carbon balance of the system. The organic loading rate (OLR) of the BES, NBES, and single-stage MF was 1103.8 mg-C/L/day, corresponding to a glucose load of 13.9 mM_glucose_/L/day. The hydraulic retention time (HRT) of all reactors was 4 days

The reactor performances were compared after operation for a period more than thrice the HRT (i.e., 12 [4 × 3] days) (Fig. 2). The rector performances were similar for the cathodic and anodic sides in the BES; therefore, the average values of the cathodic and anodic sides are explained here for the BES. Inhibition of methanogenesis, based on a lower production of methane and higher production of hydrogen, was clearer in the BES than in the NBES (Fig. 2a), although the methane content in the gas was low in both the BES (7.3%) and NBES (24.4%), considering that the methane content was greater than 60% under stable methane fermentation [12]. The hydrogen contents in the gas were 25.0 and 7.4%, corresponding to low hydrogen yields of 0.42 mM/mM_glucose_ and 0.18 mM/mM_glucose_ in the BES and NBES, respectively. An average current of 0.88 A/m^2^ per electrode was observed, corresponding to hydrogen production of 1.5 mM/day, assuming that all the current is used for hydrogen production. However, the hydrogen production in the BES was greater than that in the NBES by 3.35 (= 5.85–2.50) mM/day (> 1.5); thus, the reason for increase in hydrogen production was not electrolysis, but rather microbial activity in the BES. Theoretically, acetate and butyrate formation from glucose accompany hydrogen production, although ethanol and propionate are formed from glucose under hydrogen-neutral and hydrogen-consumption pathways, respectively [24, 25]. Accordingly, a greater amount of acetate was generated as part of the soluble metabolic products in the BES than in the NBES, and the amount of propionate and butyrate generated were similar for the BES and NBES (Fig. 2b). By contrast, more ethanol was generated in the BES than in the NBES.

Most of the residual S-TOC in the effluents of the BES and NBES was transformed into methane and carbon dioxide in the subsequent MF that was operated at an HRT of 4 days (Fig. 2c). The methane contents in the gas produced from the MFs after the application of the BES and NBES (86.7 and 85.9%, respectively) were similar and greater than that in the single-stage MF (50.0%). Thus, as expected, the total amounts of methane and carbon dioxide produced in the two-stage processes (BES → MF and NBES → MF) were higher and lower, respectively, than that in the single-stage MF (Fig. 2a).

### Increased methane production in the two-stage process of the BES and MF at a relatively high organic load

A short HRT (e.g., 0.5 h–2 days) is one of the critical parameters for inhibiting methanogenesis during dark hydrogen fermentation [12]. By shortening the HRT to 2 days at a relatively high OLR of 2207.6 mg-C/L/day (Table 1), the effect of electrochemical regulation on the reactor performance was investigated and the reactor performances of the BES and NBES were compared. This OLR corresponded to a glucose load of 27.8 mM_glucose_/L/day. In addition, the reactor performances in the second stage (i.e., MF following BES or NBES) were compared, while the MF was operated at an HRT of 4 days.

**Fig. 3:**
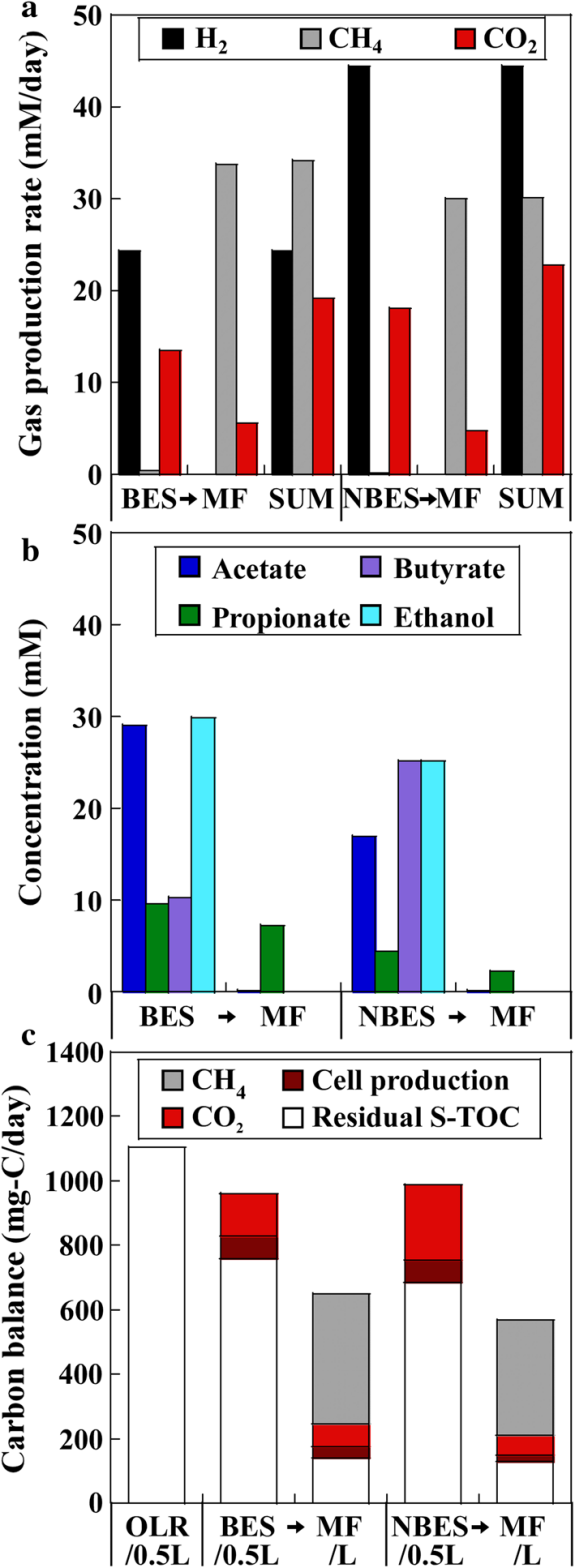
Reactor performances of two-stage processes of the bioelectrochemical system (BES) → methane fermenter (MF) and non-bioelectrochemical system (NBES) → MF. **a** Rate of gas production of hydrogen, methane, and carbon dioxide, **b** productions of soluble metabolic products. **c** Carbon balance of the system. The organic loading rate (OLR) of the BES and NBES was 2207.6 mg-C/L/day, corresponding to a glucose load of 27.8 mM_glucose_/L/day. The hydraulic retention time (HRT) of the BES and NBES was 2 days and the HRT of the second-stage MFs was 4 days

A short HRT successfully decreased the methane content in the gas produced from the BES and NBES (0.9 and 0.2%, respectively) (Fig. 3a). Thus, the hydrogen contents of the gas produced from the BES and NBES were increased to 52.1 and 53.2%, respectively. A low average current of 0.30 A/m^2^ per electrode in the BES was observed via electrode polarisation, corresponding to a hydrogen production rate of 0.5 mM/day, which was much lower than the actual rate of hydrogen production (i.e., 24.3 mM/day). In contrast to the case of a long HRT of 4 days, the hydrogen yield in the NBES (1.60 mM/mM_glucose_) was higher than in the BES (0.87 mM/mM_glucose_). Accordingly, the amount of reduced products, propionate and ethanol produced was higher and that of butyrate generation was lower in the BES than in the NBES, although the amount of acetate generated was higher in the BES than in the NBES (Fig. 3b). Similar to the result at a low OLR, most of the residual S-TOC in the BES and NBES effluents was transformed into methane and carbon dioxide (Fig. 3c). More methane was produced in the MF following the application of the BES than in the MF following the application of the NBES, and the methane contents of the gas were 85.1 and 85.6% in the MFs following the application of the BES and NBES, respectively. As a result, the amount of methane produced was higher and the amount of hydrogen and carbon dioxide produced was lower in the two-stage BES → MF process than in the NBES → MF process.

A high pH of 7–7.5 in the loaded substrate could optimise hydrogen fermentation in the batch reactors without a regulated pH, as the by-products lower the pH of the medium [16]. Thus, to eliminate the process of regulating the pH during the fermentation, an artificial glucose medium at a pH of 7.3 was loaded in the BES and NBES (Table 1). The other operational parameters were the same as those of the above operation with pH regulation (6.2–6.5) during fermentation. The results were similar to the results under pH-regulated operation. The hydrogen content in the gas produced from the BES and NBES remained high (47.6 and 47.3%, respectively) (Additional file 1: Fig. S1a). An average current of 0.34 A/m^2^ per electrode in the BES corresponded to hydrogen production of 0.6 mM/day, which was much lower than the actual hydrogen production (16.7 mM/day). The hydrogen yield in the NBES (1.00 mM/mM_glucose_) was higher than that in the BES (0.60 mM/mM_glucose_). The amount of reduced products, propionate, and ethanol generated was higher and that of butyrate was lower in the BES than in the NBES, although acetate generation was higher in the BES than in the NBES (Additional file 1: Fig. S1b). Most of the residual S-TOC in the effluents of the BES and NBES were transformed into methane and carbon dioxide (Additional file 1: Fig. S1c) and methane production was higher in the MF following the application of the BES (methane content: 86.2%) than following the application of NBES (methane content: 86.5%). The total amount of methane produced was higher and that of hydrogen and carbon dioxide was lower in the BES → MF process than in the NBES → MF process.

### Microbial 16S rRNA gene sequence analysis of the microbial consortium in the fermentation cultures

The microbial compositions developed in the fermentation cultures of the BES, NBES, subsequent MFs (← BES and ← NBES), and single-stage MF were examined. Using a combination of prokaryotic universal primers and a MiSeq platform, an average of 278,989 (± 107,052) reads was obtained for each sequencing reaction (Table 2). The number of OTUs, which can robustly estimates the species richness, was lower in the cultures in the first stage (BES or NBES) than in the second stage (MF). The alpha-diversity values (α-diversity; Chao 1, Shannon index, and Simpson index) are shown in Table 2. The Chao1 values, which indicate the species richness of the community, were lower in cultures of the first stage than in the second stage. Accordingly, the Simpson and Shannon diversity indices, which emphasise the species diversity and evenness of the consortium, were lower in the cultures of the first stage than in those of the second stage. Notably, at a short HRT (2 days) (i.e., high organic load), the number of OTUs and α-diversity values were higher in the cultures of the BES than in those of the NBES.

**Table 2:** Summary of the 16S rRNA gene sequencing data and α-diversity values in the fermentation cultures

	BES						MF (← BES)			NBES			MF (← NBES)			MF
	Cathode			Anode												
	1	2	3	1	2	3	1	2	3	1	2	3	1	2	3	4
Read count	303,658	254,520	195,455	360,162	429,089	139,885	369,111	379,933	108,290	336,686	405,213	135,923	300,358	273,447	131,346	330,753
Observed OTUs	721	549	552	760	592	456	1767	1590	1012	757	396	328	1325	1326	900	1039
Chao1	1005	829	831	1019	837	726	2347	2141	1625	994	531	522	1829	1920	1462	1382
Shannon index	3.41	2.67	3.11	3.42	2.54	3.07	5.52	5.36	5.15	3.93	1.88	2.66	5.68	5.58	5.54	4.52
Simpson index	0.83	0.71	0.80	0.84	0.69	0.80	0.96	0.95	0.91	0.88	0.54	0.76	0.96	0.96	0.96	0.88

The fermentation cultures were obtained from the BES (cathodic and anodic sides), NBES, subsequent MFs (← BES and ← NBES), and single MF. The α-diversity values included Chao1, Shannon index, and Simpson index. The numbers 1, 2, and 3, indicate the hydraulic retention time (HRT) and pH of the substrate (pH_in_) in the first stage for the two-stage process as follows; 1: 4 days and 6.5, 2: 2 days and 6.5, and 3: 2 days and 7.3. The number 4 indicates the HRT (4 days) and pH_in_ (7.7) in the single-stage MF

**Fig. 4:**
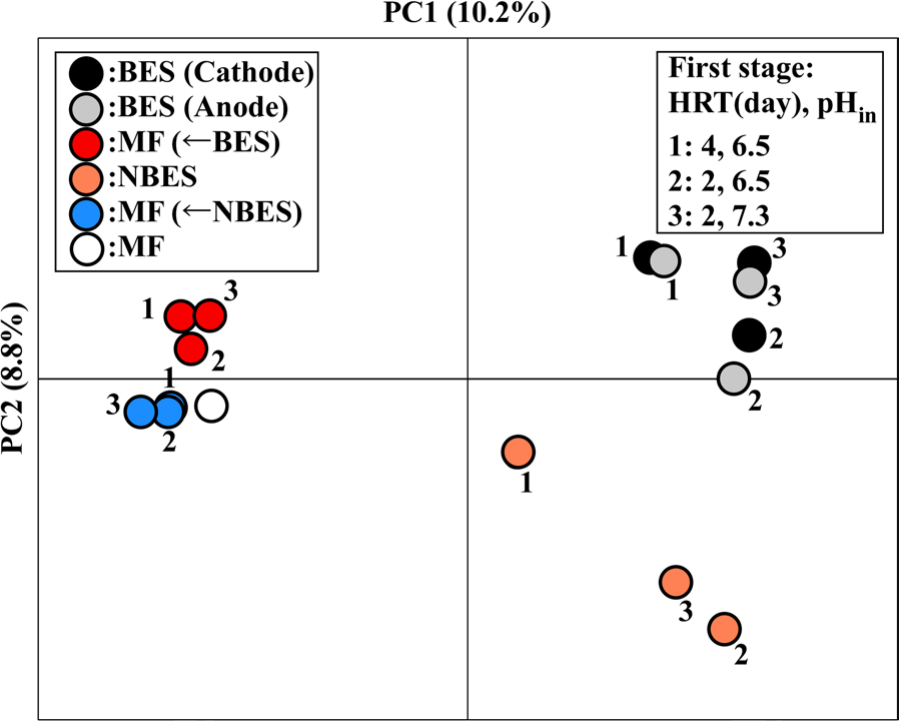
Principal coordinate plot of the 16S metagenomics data of the microbial species in the fermentation cultures on the cathodic and anodic sides of the bioelectrochemical system (BES), non-bioelectrochemical system (NBES), second-stage methane fermenters (MFs) (← BES and ← NBES), and single-stage MF. The numbers indicate the hydraulic retention time (HRT) and pH of the substrate (pH_in_) in the first stage for the two-stage process as follows: 1: 4 days and 6.5, 2: 2 days and 6.5, and 3: 2 days and 7.3

**Fig. 5:**
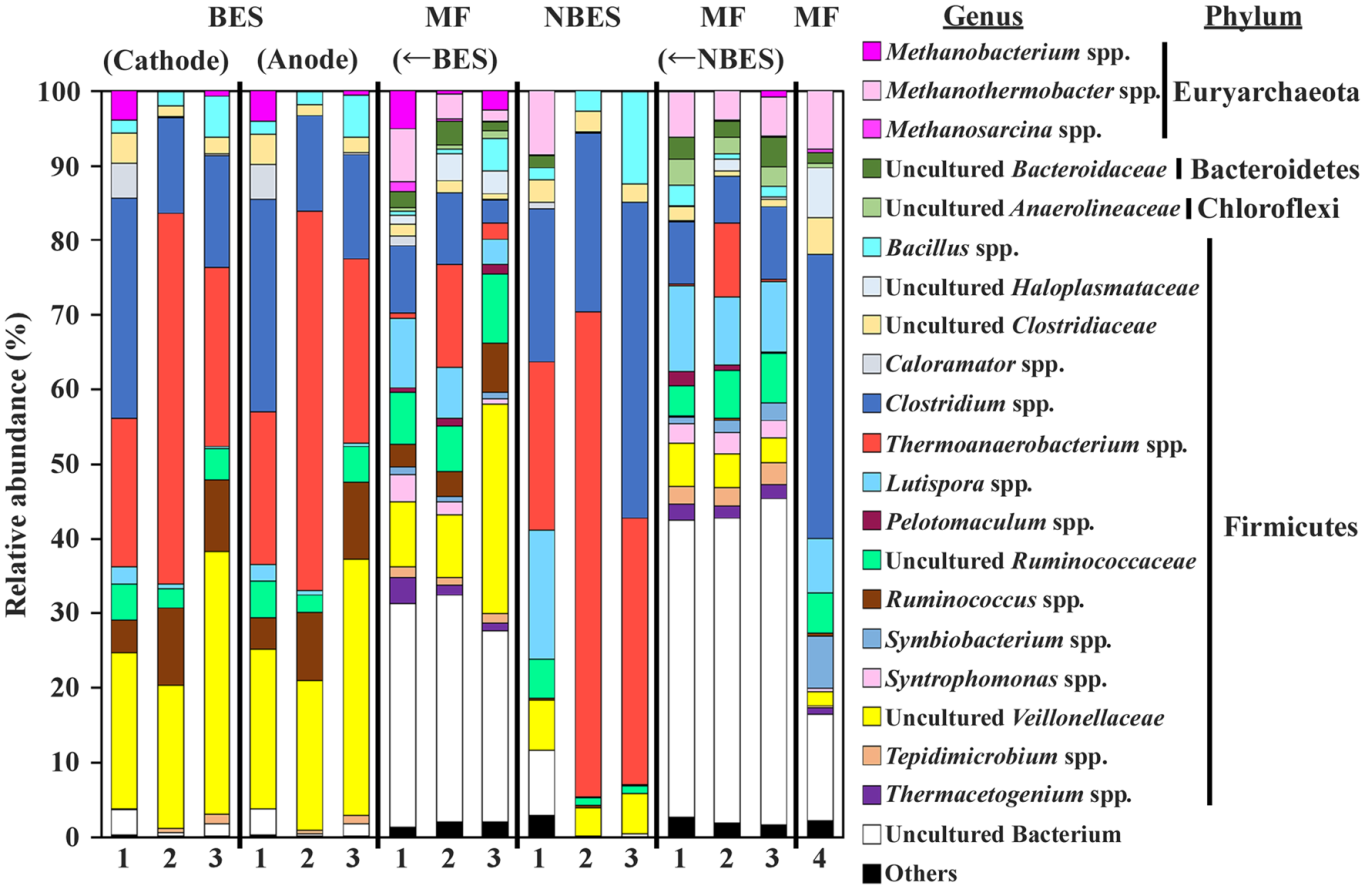
Genus-level compositional view of the microorganisms in the fermentation cultures on the cathodic and anodic sides of the bioelectrochemical system (BES), non-bioelectrochemical system (NBES), second-stage methane fermenters (MFs) (← BES and ← NBES), and single-stage MF. Genera of lower similarity (< 97%) and lower abundance (< 1.0%) were included under Uncultured Bacteria and Others, respectively. The numbers 1, 2, and 3 indicate the hydraulic retention time (HRT) and pH of the substrate (pH_in_) in the first stage for the two-stage process as follows: 1: 4 days and 6.5, 2: 2 days and 6.5, and 3: 2 days and 7.3. The number 4 indicates the HRT (4 days) and pH_in_ (7.7) in the single-stage MF

PCoA of unweighted UniFrac distances revealed that the microbial compositions differed between the BES and NBES (Fig. 4). However, the microbial compositions were similar for all MFs. The microbial composition was examined at the genus level (Fig. 5). Most of the microorganisms were assigned to four phyla (Firmicutes, Chloroflexi, Bacteroidetes, and Euryarchaeota) in all reactors. The dominant phylum was Firmicutes in the BES and NBES, consisting of mainly microorganisms belonging to the *Clostridium* and *Thermoanaerobacterium* genera, particularly in the NBES. However, the relative abundance of microorganisms belonging to *Ruminococcus* and uncultured Veillonellaceae were higher on the cathodic and anodic sides of the BES compared to that in the NBES. In all MFs, the relative abundance of microorganisms belonging to methanogens (i.e., the *Methanobacterium*, *Methanothermobacter,* and *Methanosarcina*) increased compared to thoset in the BES and NBES. In addition to methanogens, many bacterial species belonging to uncultured Bacteroidaceae, uncultured Anaerolineaceae, and the *Pelotomaculum*, *Syntrophomonas*, and *Thermoacetogenium* increased in all MFs. The relative abundance of microorganisms belonging to *Clostridium* increased in the single-stage MF process compared to the second-stage MFs.

## Discussion

Methane production in the second stage was increased owing to a change in the microbial consortium, which was driven by the application of the BES in the first hydrogen fermentation stage due to a change in the microbial consortium. The two-stage process including the BES decreased the total production of carbon dioxide under an HRT of 2 days by lowering the amount of gas in the first stage with a relatively high content of carbon dioxide and increasing the amount of gas in the second stage with a relatively low content of carbon dioxide, which contributed to a decrease in the cost of upgrading biogas to remove carbon dioxide. The number and diversity of microbial species were high owing to the low electric current in the reactor under this HRT condition, although the cell production was similar for the BES and NBES (Fig. 4). High microbial diversities can enhance resistance to environmental stresses, such as high organic loads [26].

During dark hydrogen fermentation, the upper limit of the hydrogen yield of glucose is 4 mol H_2_ per mole of hexose during acetic fermentation [Eq. (1)], while 2 mol H_2_ per mole of hexose is recorded in the butyrate pathway [Eq. (2)] [16, 25]:

1 $$ {\text{C}}_{ 6} {\text{H}}_{ 1 2} {\text{O}}_{ 6} + {\text{ 2H}}_{ 2} {\text{O }} \to {\text{ 2CH}}_{ 3} {\text{COOH }} + {\text{ 2CO}}_{ 2} + {\text{ 4H}}_{ 2} $$

2 $$ {\text{C}}_{ 6} {\text{H}}_{ 1 2} {\text{O}}_{ 6} \to {\text{ CH}}_{ 3} {\text{CH}}_{ 2} {\text{CH}}_{ 2} {\text{COOH }} + {\text{ 2CO}}_{ 2} + {\text{ 2H}}_{ 2} . $$

Thus, the amount of hydrogen produced as calculated from the concentration of acetate and butyrate in the NBES at an HRT of 2 days with a substrate pH_in_ of 6.5 and 7.3 was 42.2 mM [= 2 × (17/2) + 2 × (25.2)/2] and 35.3 mM, comparable to the actual hydrogen production of 44.4 and 27.8 mM, respectively. However, the actual amount of hydrogen produced, 24.3 and 16.7 mM, in the BES was much lower than the values calculated from the acetate and butyrate concentrations, 39.4 and 36.1 mM, at an HRT of 2 days with a substrate pH_in_ of 6.5 and 7.3, respectively. These results showed that a hydrogen-consumption reaction occured in the BES, although the ratio of hydrogen to carbon dioxide in the gas was unchanged in the BES and NBES. This was also supported by the fact that the hydrogen yield was lower in the BES (0.60–0.87 mM/mM_glucose_) than in the NBES (1.00–1.60 mM/mM_glucose_) at an HRT of 2 days, as the typical hydrogen yield ranges from 1 to 2.5 mM/mM_glucose_ [25].

Next, we considered the reason for the low hydrogen yield caused by the low electric current induced by electrode polarisation in the BES operated at an HRT of 2 days. It is reasonable that microorganisms belonging to *Bacillus*, *Clostridium*, and *Thermoanaerobacterium* were dominant in both the BES and NBES, because these organisms include typical anaerobic fermentative bacteria that convert monosaccharides into hydrogen [27–30]. *Clostridium* and *Thermoanaerobacterium* are the dominant hydrogen producers during acetate/butyrate fermentation under thermophilic conditions [24, 31]; thus, a decrease in these genera corresponds corresponded with decreased butyrate production in the BES. Interestingly, microorganisms belonging to the *Ruminococcus* species that reportedly produce ethanol in addition to hydrogen and acetic acid [32], increased in the BES. Accordingly, this corresponded with increased ethanol production in the BES. Microorganisms belonging to the Veillonellaceae family, which is known to produce propionate as a major fermentation product [33, 34], also increased in the BES. This result corresponds with increased propionate production in the BES, which contributes to hydrogen consumption [Eq. (3)]:

3 $$ {\text{C}}_{ 6} {\text{H}}_{ 1 2} {\text{O}}_{ 6} + {\text{ 2H}}_{ 2} \to {\text{ 2CH}}_{ 3} {\text{CH}}_{ 2} {\text{COOH }} + {\text{ 2 H}}_{ 2} {\text{O}} . $$

An increase in volatile fatty acids, except butyrate, led to a decrease in hydrogen production in the BES, whereas acetate production increased. One explanation for the increased acetate production irrespective of the lower hydrogen yield is that an acetogenic hydrogen-consuming reaction, homoacetogenesis [Eq. (4)] [35, 36], may occur in the BES; however, this has not been clarified

4 $$ 2 {\text{CO}}_{ 2} + {\text{ 4H}}_{ 2} \to {\text{ CH}}_{ 3} {\text{COOH }} + {\text{ 2H}}_{ 2} {\text{O}} . $$

The mechanism of the change in the structure of the microbial community is interesting. Direct electron transfer between the electrode and microorganisms [37] had a low impact in the reactions because of the low current density. At an HRT of 4 days, the BES inhibited methanogenic archaea, corresponding to the results of our previous research [15]. Previous studies have shown that the redox potential in the fermentation culture affects the growth of methanogenic archaea [38, 39]. We speculate that a high redox potential owing to the anodic reaction suppressed the growth and/or methanogenesis by methanogenic archaea [15], considering the fact that the redox potential of the anode was 0.85 V in this study. Villano et al. [40] showed that cathodic reaction increased isobutyrate production in the microbial consortium cultured in the BES with a proton exchange membrane to separate the cathode and anode. Thus, the construction of an environment with different redox potentials in the reactor could change the microbial consortium structure, leading to increased growth of microorganisms related to the *Ruminococcus* genus and Veillonellaceae family.

The species richness was higher in the second stage (i.e., MF) compared to that in the first stage, probably due to the neutral pH conditions during methane fermentation. For methanogenesis, hydrogenotrophic methanogens (i.e., *Methanobacterium* and *Methanothermobacter*) and acetoclastic methanogen (i.e., *Methanosarcina*) [12], increased in the MF cultures. It is reasonable that an increase in microorganisms related to *Pelotomaculum* and *Syntrophomonas* was observed in the MF cultures, because these microorganisms reportedly grow via syntrophy with methanogens to degrade propionate and butyrate, respectively [41, 42]. Interestingly, the microbial community structure in the fermentation cultures did not differ significantly between second-stage and single-stage MF, probably due to the retention of major microorganisms in the CFT [18, 43].

## Conclusions

We assessed the application of a BES in the first stage of a two-stage process to recover hydrogen and methane using glucose as a model organic substrate. The BES used low-cost carbon sheets and applied electric current that was low enough to prevent water electrolysis by electrode polarisation. The initial pH in the first stage was relatively high, 6.5 or 7.3, to decrease the cost of lowering the pH. The second stage included CFTs in the reactor for efficient methane generation. The effect of electrode polarisation by the BES on the suppression of methanogenesis was clear at a relatively long HRT. Moreover, the electrode polarisation changed the microbial consortium structure and metabolic patterns. Particularly at a relatively short HRT, the BES increased the species richness of the microbial consortium and the relative abundance of microorganisms related to the *Ruminococcus* genus and Veillonellaceae family, corresponding to an increase in the generation of ethanol and propionate. A decreased relative amount of microorganisms related to the *Clostridium* and *Thermoanaerobacterium* genera corresponded to a decrease in the generation of hydrogen and butyrate in the BES. In addition, a greater amount of acetate was generated in the BES. These changes were likely triggered by changing the redox potential of the electrode; however, future clarification of this mechanism is necessary. The BES reduced the amount of gas produced in first stage and increased the amount produced in the second stage. This resulted in an increase in the generation of methane and decrease in the generation of carbon dioxide in the two-stage process.

## Additional file

**Additional file 1: Fig. S1.** Reactor performances of the two-stage processes of the bioelectrochemical system (BES)→methane fermenter (MF) and non-bioelectrochemical system (NBES) →methane fermenter (MF). (a) Rate of gas production of hydrogen, methane, and carbon dioxide. (b) Production of soluble metabolic products. (c) Carbon balance of the system. The organic loading rate (OLR) of the BES and NBES was 2207.6 mg-C/L/day, corresponding to a glucose load of 27.8 mM_glucose_/L/day. The pH of the substrate was 7.3. The hydraulic retention time (HRT) of the BES and NBES was 2 days and the HRT of second-stage MFs was 4 days.

## Abbreviations

BES: bioelectrochemical system
NBES: non-bioelectrochemical system
MF: methane fermenter
rRNA: ribosomal RNA
OLR: organic loading rate
MEC: microbial electrolysis cell
CFTs: carbon fibre textiles
DSMZ: Deutsche Sammlung von Mikroorganismen und Zellkulturen
HRT: hydraulic retention time
S-TOC: soluble total organic carbon
EDTA: ethylenediaminetetraacetic acid
PCR: polymerase chain reaction
QIIME: Quantitative Insights Into Microbial Ecology
OTU: operational taxonomic unit
RDP: Ribosomal Database Project
PCoA: principal coordinate analysis
